# The Presence of Bacterial and Protozoan Pathogens in Wild Fallow Deer (*Dama dama*) from a Protected Area in Central Italy

**DOI:** 10.3390/biology14040342

**Published:** 2025-03-26

**Authors:** Valentina Virginia Ebani, Paolo Bongi, Chiara Trebino, Fabrizio Bertelloni, Giulia Cagnoli, Benedetta Bigliazzi, Marco Del Frate, Marco Apollonio, Francesca Mancianti

**Affiliations:** 1 Department of Veterinary Science, University of Pisa, Viale delle Piagge 2, 56124 Pisa, Italy; c.trebino@studenti.unipi.it (C.T.); fabrizio.bertelloni@unipi.it (F.B.); giulia.cagnoli@phd.unipi.it (G.C.); benedetta.bigliazzi@studenti.unipi.it (B.B.); francesca.mancianti@unipi.it (F.M.); 2 Centre for Climate Change Impact, University of Pisa, Via del Borghetto 80, 56124 Pisa, Italy; 3 Department of Veterinary Medicine, University of Sassari, 07100 Sassari, Italy; bongip73@yahoo.it (P.B.); delfratemarco@gmail.com (M.D.F.); marcoapo@uniss.it (M.A.)

**Keywords:** fallow deer, pathogens, *Theileria cervi*, *Anaplasma phagocytophilum*

## Abstract

Wild ruminants often harbor pathogens transmissible to other animals and humans, but their epidemiological role is not always defined for all microorganisms. In this survey, spleens, kidneys, and hearts sampled from 162 fallow deer (*Dama dama*) were subjected to molecular analyses to detect bacterial (*Anaplasma phagocytophilum*, *Borrelia burgdorferi* s.l., *Brucella* spp., *Chlamydia abortus*, *Coxiella burnetii*, *Francisella tularensis*, *Leptospira* spp.) and protozoan (piroplasms, *Neospora caninum*, *Toxoplasma gondii*) pathogens. Five (3.08%) spleens were positive for *A. phagocytophilum*, and twelve (7.40%) spleens were positive for *Theileria cervi*. The remaining pathogens investigated were not detected, and no coinfections were found. The analyzed animals do not seem to have a relevant role in the spreading of these pathogens; however, monitoring is pivotal to understand the epidemiological scenarios and take appropriate preventive measures in areas frequently visited by people.

## Simple Summary

Wild ruminants may harbor pathogens transmitted by hematophagous arthropods, as well as those transmitted via oral and/or inhalation routes. Among these microorganisms, several bacteria and protozoa may also infect humans, livestock and companion animals. In fact, wild ruminants often serve as reservoirs without showing clinical signs, whereas other mammals can develop mild or severe diseases. Wild ruminants are largely present in different areas of Central Italy, but although some studies have been carried out on the occurrence of different bacterial and protozoan pathogens in Italian deer populations, the current epidemiology of these microorganisms in deer is not fully clear because the distribution and prevalence of the pathogens are constantly changing.

## 1. Introduction

Wild ruminants, such as red deer (*Cervus elaphus*), roe deer (*Capreolus capreolus*) and fallow deer (*Dama dama*), often harbor pathogens transmitted by hematophagous arthropods, as well as agents transmitted via oral and/or inhalation routes [1,2,3,4]. Among these pathogens, several bacteria and protozoa may also be the cause of infections and diseases in human patients and domestic animals, i.e., farm and companion animals. In fact, wild ruminants often act as reservoirs without showing clinical signs, whereas other mammals can develop mild or severe diseases [3].

Wild ruminants are largely present in different areas of Central Italy; in particular, a fallow deer population live in the Regional Park Migliarino-San Rossore-Massaciuccoli, located in the northwest of Tuscany, Central Italy. From an epidemiological perspective, this protected area is of particular interest due to its proximity to the city center and the high number of visitors it receives daily [5]. Additionally, the deer population within the park exhibits a notably high density that reaches 99.8 heads/100 ha during the spring season [6]. As widely recognized, an increased ungulate population density facilitates the transmission and spread of diseases [7,8,9].

Even though some studies have been carried out on the occurrence of different bacterial and protozoan pathogens, in particular those transmitted by hematophagous arthropods, in Italian deer populations [10,11,12,13,14,15,16,17], the current epidemiology of these microorganisms in deer is not fully elucidated because the distribution and prevalence of the pathogens are constantly changing.

Therefore, the aim of the present survey was to verify the presence of bacterial (*Anaplasma phagocytophilum*, *Borrelia burgdorferi* sensu lato (s.l.), *Brucella* spp., *Chlamydia abortus*, *Coxiella burnetii*, *Francisella tularensis*, *Leptospira* spp.) and protozoan (piroplasms, *Neospora caninum*, *Toxoplasma gondii*) pathogens, most of which are transmitted by hematophagous arthropods, in fallow deer from the Regional Park.

## 2. Materials and Methods

### 2.1. Study Area

**Figure 1:**
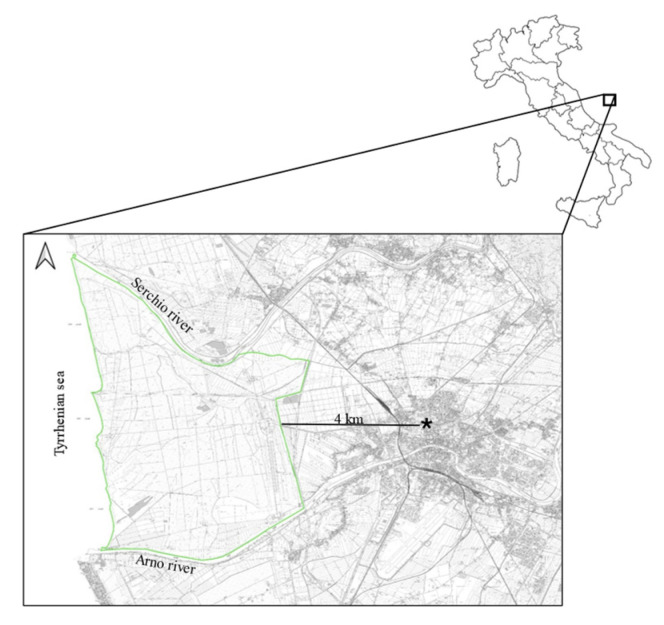
Map of the study area. Light green line represents a border of the study area, while star represents the leaning tower.

The analyzed animals lived in the San Rossore Estate, a protected area of Regional Park Migliarino-San Rossore-Massaciuccoli that covers a surface area of approximately 4950 hectares, characterized by wooded areas, wetlands and agricultural landscapes, close to the Pisa city (43°41′ N; 10°19′ E; Figure 1). The wooded areas are dominated by deciduous and coniferous forest where the predominant species are *Quercus robur*, *Quercus ilex*, *Populus alba*, *Fraxinus* spp., *Pinus pinaster*, and *Pinus pinea*. Wetlands show the presence of *Carex* spp., *Phragmites australis* and *Juncus* spp., while agricultural landscape are dominated by meadows and pasture. Fallow deer population exhibited a spring density with an estimated average of 42.8 deer/km^2^ (±8.1 SD), and in the recent past reached a peak of 99.8 deer/ km^2^ [18]. Several animal species are homed in the park: wildlife species are wild boar (*Sus scrofa*), wolf (*Canis lupus*), red fox (*Vulpes vulpes*), badger (*Meles meles*), weasel (*Mustela nivalis*), pine marten (*Martes martes*) and stone marten (*Martes foina*); domestic horse and cattle are also present. Numerous wild bird species reside in the park, which also serves as a stopover habitat for migratory birds [19].

### 2.2. Sampling

Sampling was conducted on fallow deer from July to December 2022, during maximum effort to implement the management plan. The animals were taken by gamekeepers in compliance with the management plan adopted and authorized by the park authority. Technical staff assisted the gamekeepers and conducted sampling following each fallow deer culling. Fallow deer did not show clinical signs and they did not undergo postmortem examination because they were destined for meat consumption. We supported the gamekeepers and carried out the sampling directly, between 1 and 3 h after the death of the deer.

In detail, spleen and kidney samples were collected from 162 fallow deer of which n = 25 bucks (male over 4 years old), n = 13 sores (male 2–4 y.o.), n = 11 prickets (male 1–2 y.o.), n = 29 fawns (male younger than 1 y.o.), n = 58 adult female (over 4 y.o.) and n = 29 fawns (female younger than 1 y.o.). Furthermore, from a subsample (78 deer of which n = 14 bucks, n = 11 sores, n = 11 prickets, n = 13 fawn males, n = 17 adult females, n = 12 fawn females), we also collected the hearts.

All samples were collected at the local butcher shop within the park using sterilized equipment to ensure aseptic conditions. Following killing, each fallow deer was immediately eviscerated in a refrigerated room. Using a sterilized scalpel, portions of the spleen, kidney, and heart were excised. Each sample weighed a minimum of 100 g and was collected in a sterilized plastic box. While awaiting transport to the analysis laboratory, the samples were stored in a refrigerator at a maximum temperature of 4 °C. Delivery to the laboratory occurred within 6 to 30 h after sampling.

### 2.3. Molecular Analyses

Samples were promptly submitted for DNA extraction. About 10 mg of tissue was collected from the inner spleen and myocardium; 10 mg of kidney cortex was taken after removing the renal capsule. All specimens were submitted to the DNA extraction with the DNeasy Tissue kit (Qiagen GmbH, Hilden, Germany) according to the manufacturer’s instructions; extraction controls to monitor cross-contamination of samples were included. DNA was quantified by measuring the optical density (OD) at 260 nm. Meanwhile, DNA purity was determined by calculating the ratio of absorbance at 260 and 280 nm; DNA extracts with an A260/A280 ratio of greater than 2.0 were considered of good quality and used in PCR assays. OD measurements were performed using a Nanodrop ND–1000 spectrophotometer (NanoDrop Technologies, Wilmington, DE, USA). DNAs were stored at 4 °C for 24–48 h, then used in PCR assays.

All spleens were tested for *A. phagocytophilum*, *B. burgdorferi* s.l., *Brucella* spp., *C. abortus*, *C. burnetii*, *F. tularensis*, *N. caninum*, *T. gondii*, and piroplasms. Heart samples were analyzed for *T. gondii* and *N. caninum*, and kidneys for *Leptospira* spp.

Different PCR protocols, summarized in Table 1, were employed to detect the investigated pathogens. Negative and positive controls were added in each PCR assay. Sterile distilled water was used instead of DNA in the negative control. DNA samples extracted from slides used for indirect immunofluorescent assay or bacterial cultures were included as positive controls (Table S1).

For each protocol, PCR reactions were carried out in a 25 µL final volume, containing 12.5 µL EconoTaq PLUS 2× Master Mix (Lucigen Corporation, Middleton, WI, USA), 0.3 µM of each primer, 3 µL of extracted DNA and ultrapure water to reach the final volume. All PCR amplifications were performed in an automated thermal cycler SimpliAmp™ Thermal Cycler (Applied Biosystems, Waltham, MA, USA): 95 °C for 5 min of initial denaturation followed by 40 cycles at 95 °C for 1 min; annealing (temperatures reported in Table 1) for 1 min; and 72 °C for 2 min. A final step of 10 min at 72 °C completed the reaction.

For the detection of *A. phagocytophilum* DNA, a nested PCR protocol was used [20].

For the detection of piroplasms, the first PCR protocol was used [26]; positive samples were successively subjected to a second PCR assay, amplifying a longer fragment (about 1700 bp) of the 18S rRNA [27] in order to achieve correct species identification with sequencing analyses.

All PCR products were analyzed by electrophoresis on 1.5% agarose gel at 100 V for 45 min; gel was stained with ethidium bromide and observed. SharpMass™ 100 Plus Ladder (Euroclone, Milano, Italy) was added as a DNA marker.

Successively, amplicons of piroplasms obtained with the second PCR assay were sequenced by a commercial laboratory (BMR-Genomics, Padova, Italy) because most Babesia and Theileria species are amplified using the same set of primers for their similarity in the target gene. In order to confirm the positive results, *A. phagocytophilum* amplicons obtained with the second PCR step were submitted to sequencing analysis by the same commercial laboratory.

The resulting sequences were assembled and manually corrected through visual inspection of the electropherogram using BioEdit v.7.0.2. Subsequently, they were compared with reference sequences available in GenBank using the BLAST program 2.15.0 (http://www.ncbi.nlm.nih.gov/BLAST, accessed on 12 September 2024).

## 3. Results

We collected spleen, kidney, and heart samples, with the distribution reported in Table 2 and Table 3.

All kidney samples were PCR-negative for *Leptospira* spp.

PCR for *T. gondii* and *N. caninum* carried out on all spleen and heart samples detected no positive reactions. All spleens were negative for *F. tularensis*, *Brucella* spp. *C. abortus*, *C. burnetii* and *B. burgdorferi* s.l.

Five (3.08%; 95% CI: 0.42–5.74%) spleens were positive for *A. phagocytophilum*, confirmed by the sequencing analyses of the amplicons obtained in the second PCR step. Twelve (7.40%; 95% CI: 3.37–11.43%) spleens were positive for piroplasm, and in all cases, the sequencing analyses of the amplicons obtained in both PCR assays identified *Theileria cervi*. No coinfections were detected.

## 4. Discussion

All tested fallow deer were negative for *Brucella* spp., in agreement with our recent study carried out in roe deer living in Central Italy [15]. These findings are strictly related to the Italian prophylaxis plans that have significantly reduced the circulation of brucellae; Tuscany is currently considered an officially free territory according to community legislation (EU legislation 1332 of 17 May 2024 for bovine brucellosis, EU legislation 2032 of 29 July 2024 for ovine-caprine brucellosis) [30]. Deer are mainly susceptible to *B. abortus* and *B. melitensis* infection, but they are not considered important in the epidemiology of these bacteria because they seem to act as dead-end reservoirs [31]. The limited cases of brucellosis reported in deer populations suggested this role [31,32,33], although serologically positive deer have been detected [34]. *Brucella abortus* and *B. melitensis* are pathogens responsible for severe disease in humans and animals, mainly ruminants, in which they cause reproductive disorders [32].

No fallow deer positive to *F. tularensis* were detected, in agreement with previous surveys carried out in deer [13] and other wild animals [35,36,37] in Central Italy. *Francisella tularensis* is the etiologic agent of the zoonosis called tularemia; it is a highly infectious bacterium transmitted by tick bites and via the oral/inhalation route [38]. *Francisella tularensis* has been isolated worldwide from more than 250 species, mainly lagomorphs and rodents, but also insectivores, carnivores, marsupials, ungulates, birds, amphibians, fishes, and invertebrates [38]. It is present in the Northern hemisphere, and it does not appear to be widespread in certain areas, such as Central Italy, where, however, the constant monitoring of wildlife is essential to better evaluate the risk of transmission to humans.

In this survey, all samples were *C. burnetii*-negative. This is the etiologic agent of the Q fever, a zoonotic infectious disease responsible for relevant reproductive disorders, mainly abortion, in ruminants and other mammal species [39]. Recently, *C. burnetii* DNA was found in 3/72 (4.16%) spleens sampled from roe deer in Central Italy [15], but the infection has also been documented in fallow, red, and roe deer, in different European areas [40]. The negative results of this survey may be attributed to the absence or minimal circulation of the pathogens in the selected area. This interpretation is supported by reports from technicians and park personnel, who did not observe cases of abortion in ungulates.

Similarly, the negative results for *C. abortus* are in agreement with our recent survey carried out in roe deer from Central Italy [15]. *Chlamydia abortus* is the causative agent of enzootic abortion in sheep, but it also often infects cattle; wild ruminants are known as susceptible hosts [23]. However, it is difficult to understand if these findings are related to the absence of the pathogen in the selected area or to scarce susceptibility of wild ruminants to *C. abortus*. In fact, information about chlamydiosis in deer is scant; only one case report described abortion related to *C. abortus* in a springbok antelope (*Antidorcas marsupialis*) in France [23], and further data are based on serological surveys. In particular, in Italy, the seroprevalence of 79% for *C. psittaci* was found in fallow deer [41]; the seroprevalences of 9.6% and 3.3% were observed in red deer for *C. psittaci* and *C. suis*, respectively, but no animals had antibodies to *C. abortus* and *C. pecorum* [42].

No samples had *B. burgdorferi* s.l. DNA, in agreement with a previous survey carried out in Poland, where all 74 analyzed red deer were PCR-negative for this pathogen [43]. *Borrelia burgdorferi* s.l., the causative agent of Lyme disease, is known as a tick-borne bacterium able to infect different animal species and cause disease in humans, dogs, horses and cattle [21]. The susceptibility of *O. virginianus* to *Borrelia lonestari* was demonstrated through an experimental infection; infected deer did not show overt clinical signs of disease at any time during the experiment, but they developed spirochetemia detectable by the direct examination of blood smears and/or by PCR [44].

Conversely, it has been supposed that deer are incompetent hosts for borrelia [45], and our results could be related to this aspect. In fact, borreliae are known to be sensitive to destruction by the complement system of host cervid species; the borreliacidal activity of white-tailed deer (*Odocoileus virginianus*) serum has been recently demonstrated [45]. However, few studies have been carried out to detect this pathogen in blood and tissues from deer. In Central Italy, 2/60 (3.33%) red deer and 1/72 (1.39%) roe deer were PCR-positive [13,16]. One roe deer (0.21%), among the 461 analyzed, was positive for *B. burgdorferi* s.l. in Netherland [2]. Lane et al. [46] found *B. burgdorferi* DNA in 5.12% and 20.31% of black-tailed (*Odocoileus hemionus colombianus*) in two different areas of Northern California, respectively, as did Trout-Fryxell et al. [47] in 21.2% of white-tailed deer blood samples in Arkansas (USA).

The 3.08% prevalence found for *A. phagocytophilum*, while not high compared to previous surveys [48], confirms that deer are susceptible to the pathogen and indicates the presence of this bacterium in the geographic area where animals live. The small number of positive animals did not allow for statistically evaluating the differences between age/gender classes or months of sampling. This tick-borne bacterium can infect several animal species, most of which act as asymptomatic reservoirs; other species, such as dogs, cattle and horse, often develop disease characterized by mild or severe signs [49,50]. A past investigation in fallow deer living in the same natural park detected a prevalence of 72.4% of this bacterium [48]; in addition, *A. phagocytophilum* DNA was found in 40% of red deer [13] and 59.72% of roe deer [16] from other areas in Central Italy. Many surveys have been carried out in deer populations living in different European areas; prevalences ranging from 20% to up 90% were found [1,14,51,52,53,54]. Recently, it has been supposed that *C. elaphus* and *C. capreolus* may serve as reservoirs of zoonotic *A. phagocytophilum* strains [55].

All heart and spleen samples scored negative for *N. caninum* DNA. This finding partially agrees with data from the literature. Fallow deer, in fact, have been surveyed by serological methods [56,57,58,59,60] only, and seroprevalence resulted ranged from 0% [59,60,61] to 2.9% [57]. Although in a limited study in Mexico, 2 out of 19 tested fallow deer were serologically positive [56]. Molecular analyses show a lower sensitivity when compared to serology, so our results are not surprising. However, *N. caninum* could represent a threat for *D. dama*, being reported as responsible for a fatal case of meningoencephalitis in a 3-week-old fawn [62]. Even if not zoonotic, this parasite is a leading cause of bovine abortion and stillbirth, as well as capable of causing neuromuscular disease in dogs [56], and, being both animal species present in San Rossore Park, attention should be paid.

Similarly, DNA from *T. gondii* was not found in any specimens. This finding fully agrees with the results provided by Stollberg et al. [63] who found a 0% PCR prevalence rate in 80 fallow deer from Brandeburg (Germany), with a 6.8% seroprevalence. Similarly, a 1% seroprevalence was reported in *D. dama* from Czek Republic [58]. Furthermore, serological surveys yielded seroprevalences ranging from 0% [59] to 37.4% [64], showing a large variability of this value among different populations. Interestingly, a wild ungulates community from Spain showed the highest seroprevalence rates [64,65]. Other cervids have been checked for these protozoa in our region, with different results. A previous molecular survey on the blood samples of red deer from another area of Tuscany reported a prevalence of 22% and 28% for the DNA of *T.gondii* and *N. caninum*, respectively [66], while a further recent study on the spleen specimens of roe deer showed a prevalence of 1.38% for *T. gondii* and 0% for *N. caninum* [15].

Unfortunately, serum samples of the selected animals were not available in our study, precluding the evaluation of seroprevalence rates, so the occurrence of *N. caninum* and *T. gondii*, although low, cannot be ruled out.

*Theileria cervi* was detected in 7.40% of spleen specimens as the sole piroplasm species. Also, in this case, the small number of positive animals did not allow for statistically evaluating the differences between age/gender classes or months of sampling. *Theileria cervi* has been frequently reported in cervids from Canada and the USA [67], where it is primarily transmitted by *Amblyomma americanum* [68]. The piroplasm was identified in *O. virginianus* [69] and more recently in equids from Mexico [70], as well as in cervids from Argentina and Brazil [71,72]. The parasite is rarely responsible for clinical disease, except for animals living in areas with a high deer population, starved or coinfected with other agents [63,67]. To the best of our knowledge, *T. cervi* was not reported from Italy except for in a study carried out on *Rhipicephalus sanguineus* specimens from privately owned dogs in Italy [73], indicating the occurrence of this parasite in our country.

## 5. Conclusions

The fallow deer analyzed in the present investigation lived in a protected area where many wild animals, mammals and birds, are present. In addition, the park is largely frequented by people, including owners with their dogs. Most of the investigated pathogens were zoonotic, and can infect and cause diseases in dogs as well; therefore, being aware of the presence and diffusion of these agents in a given area allows for better understanding the risks of infections for people and dogs.

The tested animals were negative for most of the pathogens investigated, suggesting that they do not have a relevant role in the epidemiology of these agents. However, deer may be important for the epidemiology of the pathogens transmitted by hematophagous arthropods. The presence of deer can provide a source of blood meals for ticks in the absence of other hosts, potentially supporting larger tick populations within an environment [74].

Therefore, monitoring the spreading of pathogens in deer populations, with a particular emphasis on zoonotic ones, is pivotal to verify the epidemiological scenarios of the microorganisms and take appropriate preventive measures in areas like this park, frequently visited by people.

## Figures and Tables

**Table 1:** Target genes, primers and annealing temperature for the PCR assays carried out to detect the DNA of each pathogen.

Pathogen	Target Gene	Primer Name	Primer Sequence (5′–3′)	Amplicons (bp)	Annealing Temperature (°C)	Ref.
*Anaplasma phagocytophilum*	*16S rRNA* (first PCR)	GE3a GE10r	CACATGCAAGTCGAACGGATTATTC TTCCGTTAAGAAGGATCTAATCTCC	932	55	[20]
	*16S rRNA* (second PCR)	GE9f GE2	AACGGATTATTCTTTATAGCTTGCT GGCAGTATTAAAAGCAGCTCCAGG	546	55	
*Borrelia burgdorferi* s.l.	*23S rRNA*	JS1 JS2	AGAAGTGCTGGAGTCGA TAGTGCTCTACCTCTATTAA	261	39	[21]
*Brucella* spp.	*bcsp31*	B4 B5	TGGCTCGGTTGCCAATATCAA CGCGCTTGCCTTTCAAGGTCTG	223	60	[22]
*Chlamydia abortus*	*pmp90/91*	pmp-F pmp-R821	CTCACCATTGTCTCAGGTGGA ACCGTAATGGGTAGGAGGGGT	821	63	[23]
*Coxiella burnetii*	*IS1111*	Trans-1 Trans-2	TATGTATCCACCGTAGCCAGT CCCAACAACACCTCCTTATTC	687	64	[23]
*Francisella tularensis*	*TUL4*	TUL4-435 TUL4-863	TCGAAGACGATCAGATACCGTCG TGCCTTAAACTTCCTTGCGAT	400	60.5	[24]
*Leptospira* spp.	lipL32 [pathogenic leptospirae]	LipL32–45F LipL32–286R	AAGCATTACCGCTTGTGGTG GAACTCCCATTTCAGCGA TT	242	58	[25]
Piroplasms	*18S rRNA*	Mic 1 Mic 2	GTCTTGTAATTGGAATGATGG CCAAAGACTTTGATTTCTCTC	560	50	[26]
	*18S rRNA*	Crypto F Crypto R	AACCTGGTTGATCCTGCCAGTAGTCAT GAATGATCCTTCCGCAGGTTCACCTAC	1700	65	[27]
*Neospora caninum*	*Nc5*	NP21 NP6	CTCGCCAGTCAACCTACGTCTTCT CCCAGTGCGTCCAATCCTGTAAC	337	63	[28]
*Toxoplasma gondii*	*B1*	B1outF B1outR	GGAACTGCATCCGTTCATGAG TCTTTAAAGCGTTCGTGGTC	193	57	[29]
		B1inF B1inR	TGCATAGGTTGCAGTCACTG GGCGACCAATCTGCGAATACACC	96	62.5	

**Table 2:** Distribution in relationship to the sex and age classes of fallow deer sampled for spleen and kidney.

Sex/Age Class	July	August	September	October	November	December	TOTAL
Buck	5	0	11 (1 *, 1 **)	2 (1 **)	1	6 (1 **)	25
Sore	1	0	5	2	2	3	13
Pricket	5	0	0	2	4 (1 **)	0	11
Adult female	3	0	7	16 (1 *, 2 **)	17 (1 *, 1 **)	15 (2 **)	58
Fawn male	0	2	3	5 (1 **)	6 (1 *)	13	29
Fawn female	0	0	1	7	9 (1 **)	12 (1 *, 1 **)	29
TOTAL	14	2	27	34	39	49	165

Legend. *: number of animals positive for *Anaplasma phagocytophilum*; **: number of animals positive for *Theileria cervi*.

**Table 3:** Distribution in relationship to the sex and age classes of fallow deer sampled for heart.

Sex/Age Class	July	August	September	October	November	December	TOTAL
Buck	3	0	5	0	0	6	14
Sore	1	0	5	2	1	2	11
Pricket	5	0	0	0	2	4	11
Adult female	3	0	0	4	4	6	17
Fawn male	0	2	0	0	1	10	13
Fawn female	0	0	0	0	1	11	12
TOTAL	12	2	10	6	9	39	78

## Data Availability

All data are reported in the manuscript.

## Supplementary Materials

The following supporting information can be downloaded at: https://www.mdpi.com/article/10.3390/biology14040342/s1, Table S1: Positive controls included in the PCR assays.
