# Supplementary material for: The Presence of Bacterial and Protozoan Pathogens in Wild Fallow Deer (Dama dama) from a Protected Area in Central Italy
Source: Biology (Basel). 2025 Mar 26;14(4):342. doi: 10.3390/biology14040342 (PMC12024986; doi:10.3390/biology14040342)
Supplement: Supplementary file 1 [file biology-14-00342-s001.zip › biology-3515541-supplementary.pdf]

Table S1. Positive controls included in the PCR assays

**Pathogen**	**source**
Anaplasma phagocytophilum	IFAT slide (Fuller Laboratories, Torrance, Fullerton, CA, USA)
Borrelia burgdorferi s.l.	IFAT slide (Fuller Laboratories, Torrance, Fullerton, CA, USA)
Brucella spp.	*B. ovis* culture
Chlamydia abortus	IFAT slide (Fuller Laboratories, Torrance, Fullerton, CA, USA)
Coxiella burnetii	IFAT slide (Fuller Laboratories, Torrance, Fullerton, CA, USA)
Francisella tularensis	IFAT slide (Fuller Laboratories, Torrance, Fullerton, CA, USA)
Leptospira spp.	*L. interrogans* culture
Piroplasms	*Babesia caballi/Theileria equi* IFAT slide (Fuller Laboratories, Torrance, Fullerton, CA, USA)
Neospora caninum	IFAT slide (Fuller Laboratories, Torrance, Fullerton, CA, USA)
Toxoplasma gondii	IFAT slide (Fuller Laboratories, Torrance, Fullerton, CA, USA)
